# Use of Proton Pump Inhibitors and Risk of Fracture in Adults: A Review of Literature

**DOI:** 10.7759/cureus.49872

**Published:** 2023-12-03

**Authors:** Yubraj Paudel, Beenish Najam, Heet N Desai, Janan Illango, Kofi D Seffah, Mahendar Kumar, Namballa Naveen, Vamsi Krishna Pachchipulusu, Sai Sri Penumetcha

**Affiliations:** 1 Internal Medicine, California Institute of Behavioral Neurosciences & Psychology, Fairfield, USA; 2 Research, California Institute of Behavioral Neurosciences & Psychology, Fairfield, USA; 3 Internal Medicine, Piedmont Athens Regional Medical, Athens, USA; 4 Anaesthesia, Our Lady of Lourdes Hospital, Drogheda, IRL; 5 Emergency Medicine, Steel Authority of India (SAIL) Hospital, Dhanbad, IND; 6 Cardiology, California Institute of Behavioral Neurosciences & Psychology, Fairfield, USA

**Keywords:** adverse effects of medication, s: osteoporosis, bone mineral density, fragility fractures, proton pump inhibitors (ppis)

## Abstract

Proton pump inhibitors (PPIs) are commonly used medications for various gastrointestinal disorders and are reported to be associated with bone fractures. A literature review was performed, which showed PPI to be associated with a shorter time to first fracture in adults aged 25 or older. There was an overall increased risk of fractures with PPI use in adults; however, such risk was not significantly higher in women over 80 years of age and adult patients with rheumatoid arthritis. In healthy adult males aged 18-50 years, PPI use was not associated with significant changes in calcium and bone metabolism with PPI use. The lack of increased risk among elderly women aged more than 80 and rheumatoid arthritis patients raises the possible confounding or effect modification by factors that affect the fracture risk with PPI use. We concluded that although observational studies show an increased risk of fractures with PPI use, warranting their use with caution in some patients, experimental evidence explaining the risk is still lacking.

## Introduction and background

The primary target for the treatment of gastric acid-related diseases with proton pump inhibitors (PPIs) is hydrogen-potassium adenosine triphosphate (H+/K+ ATPase) in the secretory canaliculus of the parietal cell [1]. PPIs are activated by the acidic potential of hydrogen (pH) and bind to cysteine residues on the luminal side of the adenosine triphosphate (ATPase) and suppress the final step of acid secretion, which results in a more effective acid suppression compared to histamine receptor antagonists [2]. PPIs are widely used for peptic ulcer disease, gastroesophageal reflux disease, dyspepsia, and other gastrointestinal conditions. They are reported to be associated with an increased risk of hip, spine, and total fractures; however, the causative role of PPI in osteoporosis is still unclear [3-5]. PPIs are used for both short-term (from two to eight weeks) and long-term (over eight weeks), either continuously or intermittently, as per the nature of the disease. Prolonged intake can lead to structural changes like parietal cell hypertrophy and hyperplasia, as well as functional changes like hypergastrinemia and decreased somatostatin levels as a consequence of potent acid suppression [6]. A decreased absorption of calcium due to hypochlorhydria, impaired osteoclast function, and elevated homocysteine secondary to decreased absorption of folate and vitamin B12 are the mechanisms implicated in non-osteoporotic and osteoporotic fractures [7]. Although many studies are conducted regarding the risk of bone fractures with PPI use, more studies are needed to define their action on the bone more clearly [8]. We reviewed the risk of bone fracture with PPI use among the adult population to gain new insight into the association that could be helpful in formulating clinical guidelines regarding the use of PPI use. 

## Review

Methods

**Figure 1:**
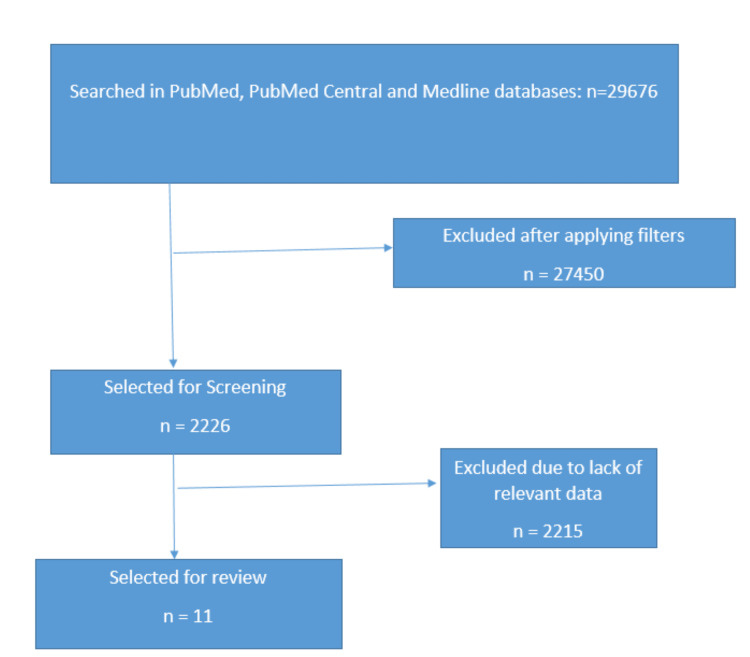
Selection of articles

We searched PubMed®, PubMed Central®, and Medline® databases using keywords "proton pump inhibitors" OR "omeprazole" OR ("proton pump inhibitors/adverse effects"[Majr] OR "proton pump inhibitors/toxicity"[Majr]) AND "bone fractures" OR "fractures, bone/etiology"[Majr]. A total of 29,676 articles were found. A total of 2226 articles were screened after using filters. We included full-text articles, books and documents, case reports, clinical studies, clinical trials, clinical trials phase IV, comparative studies, corrected and republished articles, government publications, meta-analyses, multicenter studies, observational studies, pragmatic clinical trials, randomized controlled trials, reviews, and systematic reviews in the English language published in the last 10 years prior to 13th of January, 2023 that included studies in humans aged ≥18 years. We excluded articles lacking relevant data, animal studies, and those failing to fulfill inclusion criteria as per PRISMA guidelines [9]. A total of 11 articles were selected for detailed review and analysis (Figure 1).

Results

A systematic review and meta-analysis by Hussain et al. showed an increased risk of hip fracture with PPI use even when stratified by calcium adjustment and the duration of PPI use in adults (pooled and adjusted RR: 1.26, 95% CI: 1.17-1.35, p<0.0001) [3]. A prospective matched controlled study by Sharara et al. in healthy adult males aged 18 to 50 years showed no measurable effect in calcium or bone metabolism after 12 weeks of PPI use as evidenced by a lack of significant changes in serum calcium, vitamin D, osteocalcin, and parathyroid hormone (PTH) levels [4]. In a prospective multicenter double-blind study by Hansen et al. in healthy postmenopausal women aged 45 to 75, the use of dexlansoprazole or esomeprazole for 26 weeks showed no clinically significant changes in bone mineral density (BMD), 24-hour urinary excretion of magnesium and calcium, serum PTH, phosphorus, calcium, magnesium, and 25-hydroxy-vitamin D. The markers of bone turnover procollagen type 1 N-terminal propeptide (P1NP) and C-terminal telopeptide of type 1 collagen (CTX) were significantly elevated but remained within the normal range [5]. A Canadian multicenter cohort study over 10 years done by Fraser et al. in adults 25 years or older showed that PPI use was associated with a shorter time to first traumatic fracture when adjusted to multiple risk factors, including femoral neck bone density (HR: 1.40, 95% CI: 1.11-1.77, p=0.004) [10]. In a population-based case-control study by Freedberg et al. among the population aged four to 29 years, young adults aged 18-29 years were at increased risk of fracture with PPI exposure (adjusted OR: 1.39, 95% CI: 1.26-1.53) [11]. In a retrospective cohort study in Swedish women by Lauppe et al., PPI use increased the risk of fractures among females aged 60-79 with prior risk factors for fracture (HR: 1.2, 95% CI: 1.1-1.3, p ≤ 0.05) but not in the general population of similar age; however, in females aged 80 or higher, there was no further increase in the risk of fractures regardless of prior risk factors [12]. In a retrospective study done by Lyu et al. among kidney transplant recipients (KTRs), the use of PPIs did not increase the odds of significant bone mineral density (BMD) loss in the hip and spine [13]. A systematic review and meta-analysis on medications as a risk factor for fragility fractures by Mortensen et al. showed that PPIs increase the odds of fragility fractures, especially in hip fractures (OR: 1.41, 95% CI: 1.16-1.71, p<0.001); however, the duration of PPI use was not taken into consideration [14]. In patients with rheumatoid arthritis, a longitudinal prospective observational study done by Ozen et al. showed no further increased risk of fractures with PPI use (adjusted HR: 0.92, 95% CI: 0.80-1,06) [15]. A United Kingdom-based primary care database-related retrospective matched cohort study conducted by Zirk-Sadowski et al. in patients aged 60 or above showed an overall increased risk of fracture even when accounted for prior fragility fractures (prior event rate ratio adjusted HR: 1.27, 95%CI: 1.16-1.34) [16]. A randomized controlled trial by Itoh et al. in women with low bone mineral density aged over 50 showed a significant increase in bone mineral density when bisphosphonates were used with PPIs compared to bisphosphonates alone after nine months of use. However, this study lacked a long-term follow-up, and the effects were attributable to a probable increase in gastrointestinal absorption of bisphosphonates [17]. The results of the literature review are summarized in Table 1.

**Table 1:** The results of the literature review PTH - parathyroid hormone; PPI - proton pump inhibitors; HR - hazard ratio; OR - odds ratio; BP - bisphosphonate; H2RA - histamine-2 receptor antagonists; RR - relative risk; DXA - dual X-ray absorptiometry

Author, publication year	Type of the study	Number of participants	Age	Results
Hussain et al., 2018 [3]	Systematic review and meta-analysis of case-control and cohort studies	1,270,418	>18 years	RR: 1.26, 95% CI: 1.17-1.35, p<0.00001
Sharara et al., 2013 [4]	Prospective matched controlled study	58	18-50 years	No significant change in serum calcium, phosphorus, PTH, 25-hydroxy vitamin D, osteocalcin, C-terminal cross-linked telopeptides of type I collagen (p>0.05) after 12 months of therapy
Hansen et al., 2019 [5]	Prospective multicenter double-blind study	115	45-75 years	No significant differences in changes in markers of bone turnover with PPI use
Fraser et al., 2013 [10]	Cohort study	9,423	≥25 years	Adjusted HR for fracture 1.40, 95% CI: 1.11-1.77, p=0.004
Freedberg et al., 2015 [11]	Case-control study	219,305	18-29 years	Adjusted OR for the risk of fracture 1.39, 95% CI: 1.26-1.53 with a dose-response effect (p<0.001 for the trend)
Lauppe et al., 2019 [12]	Retrospective cohort study	26,655 (20,398 general population, 6,257 DXA group (with prior fracture risk factors)	60-79	HR: 1.1, 95% CI: 1.0-1.1 in the general population; HR: 1.2, 95% CI: 1.1-1.3 in the DXA group
		14,455 (12,755 general population, 1700 DXA group (with prior fracture risk factors)	80+	HR: 1.0, 95% CI: 1.0-1.1 in the general population; HR: 0.9, 95% CI: 0.8-1.1 in the DXA group
Lyu et al., 2020 [13]	Retrospective study in kidney transplant recipients	1774 (1478 PPI users and 296 H2RA users)	Average age of PPI users: 51.7 years; H2RA users: 45.7 years	Decreasing slope of hip T-score for PPI vs. H2RA users: adjusted OR: 1.3, 95% CI: 0.8-1.9; decreasing slope of spine T-score for PPI vs. H2RA users: adjusted OR: 1.2, 95% CI: 0.8-1.8 (outcomes measured three months after transplant, which remained statistically similar six months after transplant)
Mortensen et al., 2020 [14]	Systematic review and meta-analysis	352,366 (297,488 controls and 54,878 cases)	Average age ≥43 years	OR: 1.29, 95% CI: 1.13-1.47, p<0.001
Ozen et al., 2019 [15]	Longitudinal prospective observational study	4963	≥40 years	Adjusted HR: 0.92, 95% CI: 0.80-1.06
Zirk- Sadowski et al., 2017 [16]	Retrospective cohort study	172,938 (86,469 treatment group and 86,469 control group)	60-74 years	Adjusted HR: 1.35, 95% CI: 1.13-1.59
			75-84 years	Adjusted HR: 1.22, 95% CI: 0.96-1.39
			85+ years	Adjusted HR: 1.11, 95% CI: 0.86-1.41
Itoh et al., 2013 [17]	Randomized controlled trial	180	>50 years	% increase in bone mineral density (average±SD), p<0.05 in BP group: 12.4±19.6, in PPI+BP group: 24.6±27.4
				% decrease in bone-specific alkaline phosphatase (average±SD), p<0.01 in BP group: 31.9±26.0, in PPI+BP group: 16.4±28.1

Discussion

Proton pump inhibitors are commonly used medications to treat gastric acid-related disorders and act on H+/K+ ATPase channels in gastric parietal cells [18]. While PPIs are mostly used for a short duration, their long-term use has increased in conditions such as gastroesophageal reflux disease (GERD) and in patients on non-steroidal anti-inflammatory drugs (NSAID) or aspirin therapy. With the widespread use of these medications, there is a concern over their consequences [19]. Prolonged use of PPIs is associated with side effects such as chronic renal disease, micronutrient deficiencies (vitamin B12, iron, magnesium), osteoporotic fractures, pneumonia, and Clostridioides difficile infections. PPIs possibly inhibit vacuolar-type ATPase (V-ATPase) in osteoclasts, leading to reduced bone turnover and causing a detrimental effect on bone [20].

The higher incidence of bone fracture is also correlated with the effect of PPIs on calcium metabolism, potentially causing calcium malabsorption as calcium absorption is remarkably decreased in the presence of hypochlorhydria. This can reduce blood calcium, which alters bone formation and stimulates osteoclastic bone resorption, resulting in decreased bone mineral density [20,21]. However, this association of gastric acid suppression and reduced bone mineral density causing fractures due to calcium malabsorption is still debatable [22]. PPI-induced vitamin B12 malabsorption may elevate homocysteine levels, which can weaken bone by inhibiting lysyl oxidase, an enzyme responsible for collagen cross-linking, thereby reducing the quality of collagenous bone matrix [23]. Elevated serum gastrin levels caused by long-term gastric acid suppression can increase parathyroid hormone production, stimulating bone resorption [24].

We found inconsistent associations of bone fractures with the use of proton pump inhibitors, which varied among different patient populations in adults according to their age group, underlying medical conditions, and prior risk factors for fractures. Although most of the observational studies show an increased risk of fractures with PPI use, the lack of change in bone mineral density in kidney transplant recipients and healthy postmenopausal women, lack of increased risk of fractures among women older than 80 years and patients with rheumatoid arthritis suggest the possibility of other variables, such as concurrent use of glucocorticoids or preexisting osteopenia related to the disease itself. We suggest the need for further study stratified by age, comorbidities, dose, duration of PPI use, and other risk factors for osteoporosis and fractures that may help to formulate clear guidelines regarding the use of PPIs in people vulnerable to fractures. A more detailed and larger study, possibly eliminating confounders, can provide better insight into the role of PPIs in bone fractures, whether causative or merely an association. Furthermore, more experimental studies could provide insights into the causative role of PPIs on fractures.

## Conclusions

Many observational studies showed that PPI use was significantly associated with increased bone fractures in adults; however, this association does not seem to be caused by a significant alteration in bone metabolism. This is evidenced by a lack of significant change in serum calcium and parathyroid hormone (PTH) levels as well as maintenance of normal levels of markers of bone turnover in controlled trials in healthy adult males and healthy postmenopausal females. There was no further increased risk of fractures with PPI use in general female populations aged 60 or above; however, the risk was significant among the age group 60-79 with prior risk factors for fracture. Similarly, there was no significant increase in risk with PPI use in the age group 80 or older. There was no association of loss of bone mineral density with PPI use in kidney transplant recipients and healthy postmenopausal females. PPIs did not further increase the risk of fractures in rheumatoid arthritis patients.
